# *Gloriosa superba* poisoning mimicking an acute infection- a case report

**DOI:** 10.1186/s40360-015-0029-6

**Published:** 2015-09-28

**Authors:** Ranjan Premaratna, Mindu S. Weerasinghe, Nuwan. P. Premawardana, H Janaka de Silva

**Affiliations:** Department of Medicine, Faculty of Medicine, University of Kelaniya, Ragama, Sri Lanka; University Medical Unit, Teaching Hospital Ragama, Ragama, Sri Lanka

**Keywords:** Gloriosa superba, Hair loss, Alopecia, Febrile illness, Poisoning

## Abstract

**Background:**

*Gloriosa superba* (GSb) is a highly poisonous plant and its toxicity is due to anti-mitotic effects of constituents such as colchicine and gloriosine on rapidly proliferating cells. Poisoning is known to cause very rapid and severe clinical manifestations due gastro intestinal, neurological, cardiac and bone marrow toxicity.

**Case presentation:**

A young male presented with an acute onset febrile illness associated with diarrhoea, confusion, haematuria and aggressive behavior of 4 days duration. He developed subconjunctival haemorrhages, bleeding gums, neck stiffness, bilateral papilloedema, tender hepatomegaly and features suggestive of subacute intestinal obstruction. He had progressive reduction in white cell counts, platelets and derrangements in liver functions. The illness mimicked acute severe leptospirosis or dengue. On day 9 of illness he started to loose his hair and was totally alopecic by day 14. At this stage of illness, possibility of GSb poisoning was suspected. He admitted the act of self harm after repeated questioning.

**Conclusion:**

His presentation mimicked an acute severe tropical febrile illness such as leptospirosis or dengue until he started to loose his hair. Therefore we feel that Clinicians practicing in tropical setting where Gloriosa superba is endemic should be aware of its clinical presentations and should always consider the possibility of ingestion of *Gloriosa superba* when the patient has pancytopenia and develops shedding of hairs which results in total alopecia in a case of unexplained gastroenterocolitis, rather investigating.

## Background

The active principle constituents of *Gloriosa superba* (GSb) include highly active alkaloids such as colchicine, gloriosine, superbrine (a glycoside), chelidonic acid and salicylic acid [1, 2] All parts of the plant, especially the tubers, are extremely poisonous [1, 2]. Ingestion of tubers results in severe poisoning in humans [1–3]. Mode of poisonous action is attributed mainly to colchicine and gloriosine for their anti-mitotic activity that arrests mitosis in metaphase [2, 3]. Cells with high turnover and high metabolic rate such as intestinal epithelium, hair follicle, bone marrow cells, etc. are highly susceptible to the toxic effects of GSb [2, 3]. Lethal dose is about 6 mg/Kg body weight and the fatal period following ingestion is about 12–72 hrs [1]. Acute manifestations of poisoning appear within 2–6 hrs of ingestion and the clinical profile includes burning pain in mouth, nausea progressing to severe gastroenteritis with diarrhoea and vomiting. Later it progresses to haemodynamic instability, delirium, loss of consciousness, convulsions, respiratory distress, coagulopathy, renal failure or multi-organ failure and progressive polyneuropathy [4] that occur within 12–36 hrs [3]. Severe cardiotoxicity following GSb poisoning has been previously documented [5]. Fatal complications that lead to death include hemorrhagic complications, multi-organ failure and infective complications [3]. Severe hair loss is a well recognised feature of GSb poisoning [6].

GSb poisoning has been reported from Sri Lanka and South India. A retrospective study conducted on poisoning in 1990 in the western Sri Lanka revealed that it was responsible for 44 % of plant poisonings with a 15 % case fatality rate [7]. We report a case of GSb poisoning who presented mimicking an acute infection.

## Case presentation

**Fig. 1:**
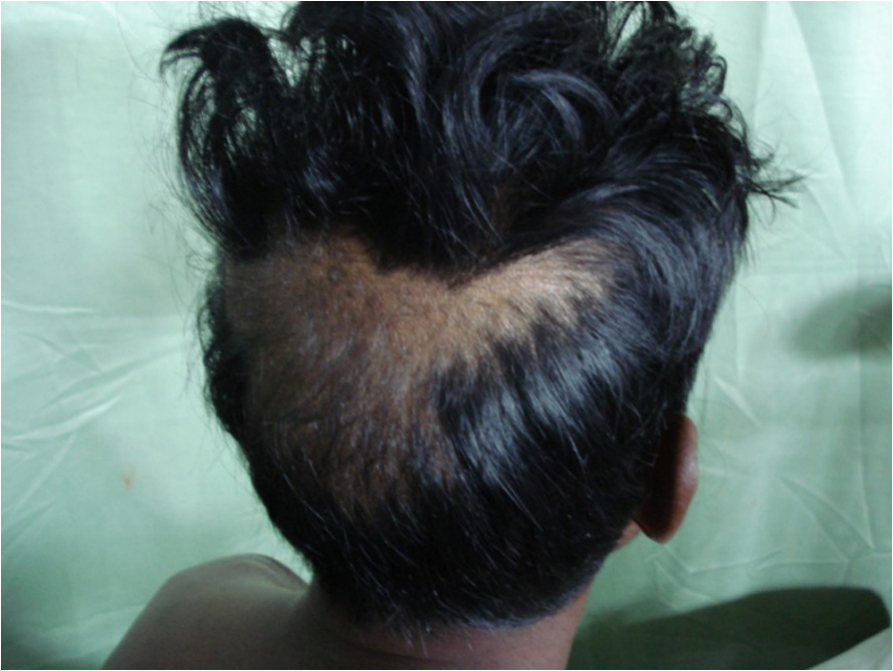
Hair loss on day 9

**Fig. 2:**
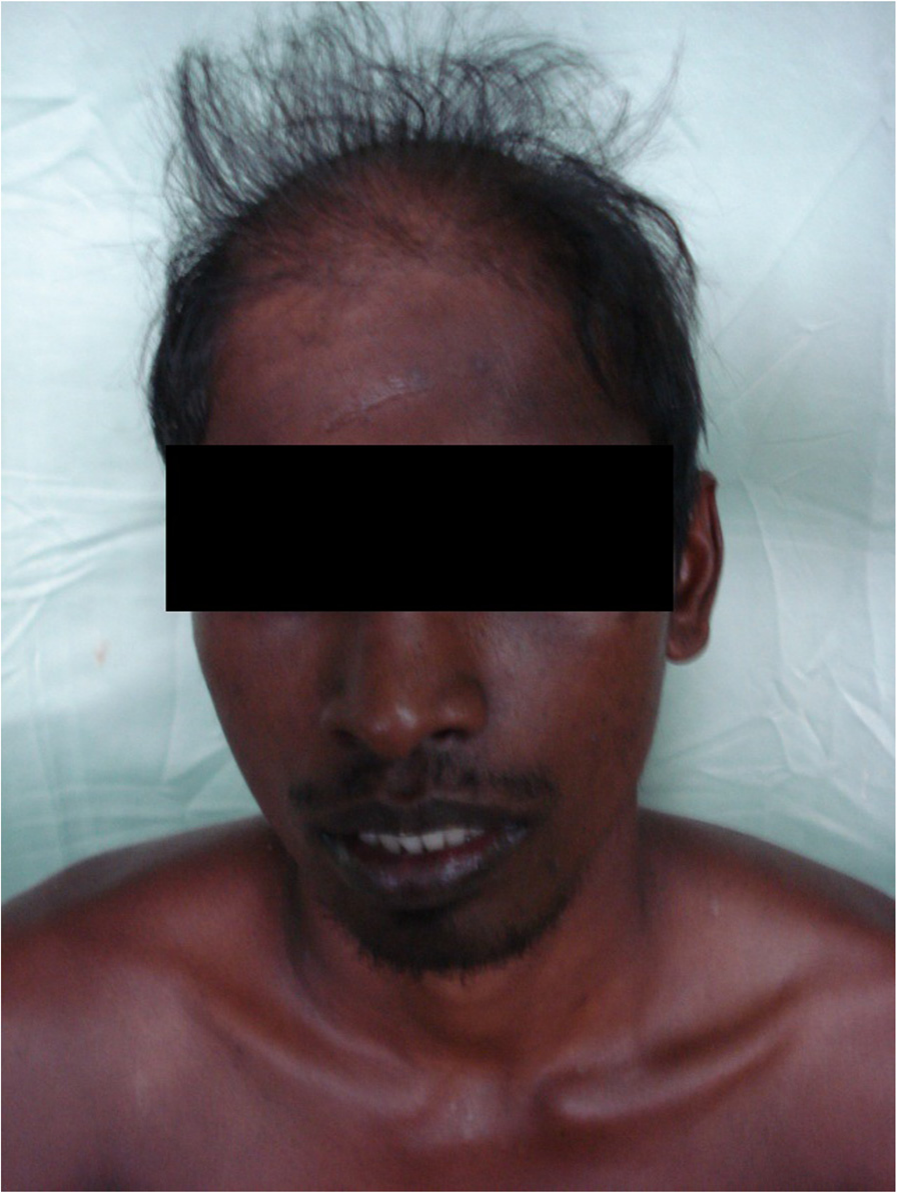
Hair loss on day 10

**Fig. 3:**
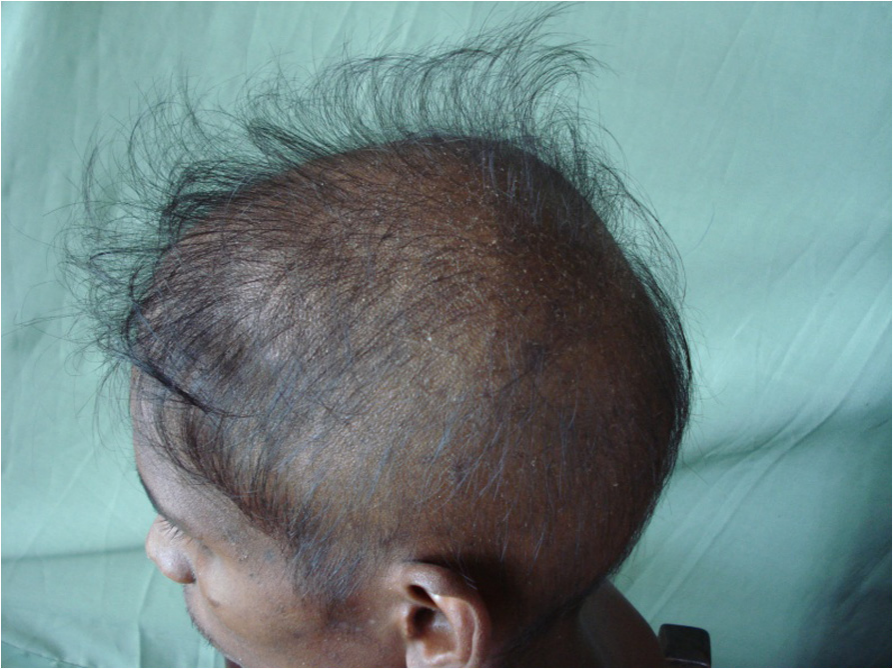
Hair loss on day 11

**Fig. 4:**
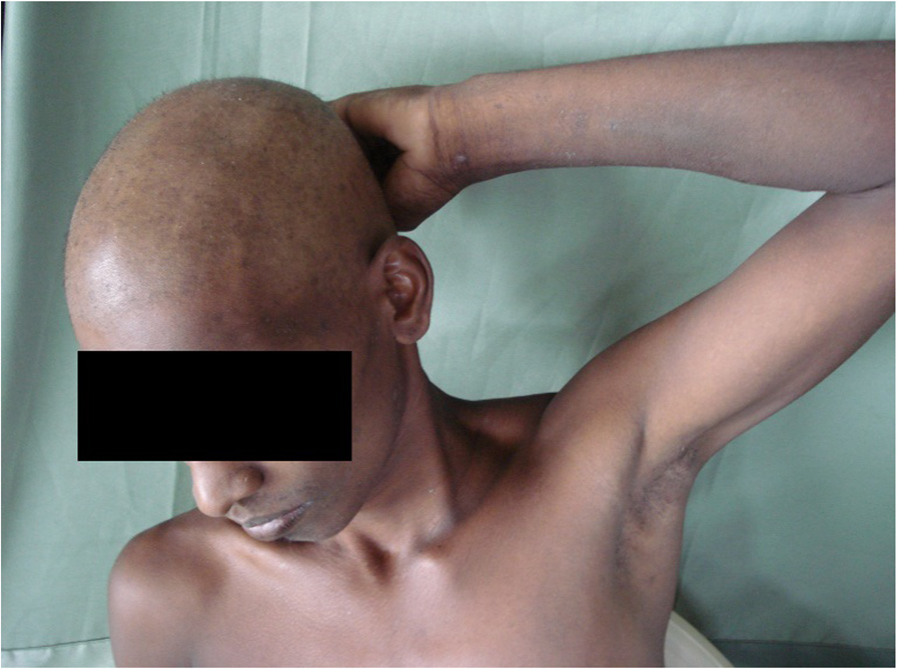
Hair loss on day 14

A 26-year-old male was admitted to the Professorial Medical Unit, Colombo North Teaching Hospital, Ragama, Sri Lanka with fever, confusion, haematuria and aggressive behavior of 4 days duration. At the onset of illness, he had vomiting and watery diarrhoea without fever, bodyaches, malena or haematemesis. He had been treated at a local hospital for a possible viral gastroenteritis or a food poisoning. During current admission, he was febrile (103 °F) and had left subconjunctival haemorrhages and bleeding gums. He was not pale and anicteric. There was neck stiffness and bilateral papilloedema but no focal neurological signs. There was a 2 finger tender hepatomegaly with no free fluid in the abdomen. The rest of system examination was normal. Investigations within the first 48 h of his acute illness revealed polymorpho-nuclear-leucocytosis with thrombocytopenia (WBC 13,000/mm^3^, neutrophils 76 %, lymphocytes 32 %, Platelet 86,000/mm^3^) and urine analysis showed protein100mg with pus cells 15–20 and red cells 150–200 per high power field. During current admission his WBC 4200/mm^3^, neutrophils 33 %, lymphocytes 56 %, platelets 45,000/mm^3^, blood urea 38 mg/dl, serum creatinine 1.1 mg/dL, serum Sodium 132 mEq/L, Potasium 3.6 mEq/L, AST 1318U/L, ALT 365U/L, serum billirubin 2.8 mg/dl, INR 1.2, CPK 574U/L, ESR 80 mm/1^st^hour and CRP 96 mg/dl. EEG was normal. Non contrast CT brain showed mild cerebral oedema. ECG was normal. 2-D echocardiogram was normal. Chest radiogram revealed right lower zone patchy opacification. USS-abdomen showed mild hepatomegaly and distended bowel loops with sluggish movements. Cerebrospinal fluid analysis was not carried out due to evidence of cerebral oedema. A probable diagnosis of acute leptospirosis, atypical pneumonia or severe dengue with encephalitis and hepatitis was considered and was commenced on empirical treatment with intravenous ceftrioxone and ciprofloxacin. Although he became afebrile over the next 48 h, the icterus progressed with rising serum bilirubin. On day 7 he had gaseous distension of the abdomen with sluggish bowel sounds and constipation suggestive of subacute intestinal obstruction. By this time, his haemoglobin dropped to 9 g/dl with granulocytopaenia (WBC 1900/mm^3^ N 20 %, L-75 %) and thrombocytopenia (Platelets 6000/mm^3^). Blood picture showed leukopaenia with few reactive lymphocytes and thrombocytopaenia suggestive of complicated acute dengue fever. On day 9, patient started to loose his hair (Fig. 1) and it progressed over the next few days [day 10 (Fig. 2) day 11 (Fig. 3)] and became totally alopecic by day 14 (Fig. 4). His Dengue IgM antibody, Leptospira haemagglutination test, mycoplasma antibodies, rickettsial antibodies were negative. The rapid hair loss together with the clinical picture suggested the possibility of GSb poisoning as he had been quite healthy prior to this acute illness. He had never been on any cytotoxic drugs and did not have any treatment for haematological or systemic malignancy or treatment with colchcine for gout, which were the other likely possibilities for rapid hair loss. He was managed symptomatically with supportive treatment and with intravenous cefotaxime. He showed gradual improvement in his illness with progressive rise in blood counts over the next two weeks. Later he admitted that he consumed two tubors of GSb as an act of deliberate self harm over a family dispute, the night before he felt ill. After one month of discharge, he had some evidence of hair regrowth however he was lost to follow up there after.

**Fig. 5:**
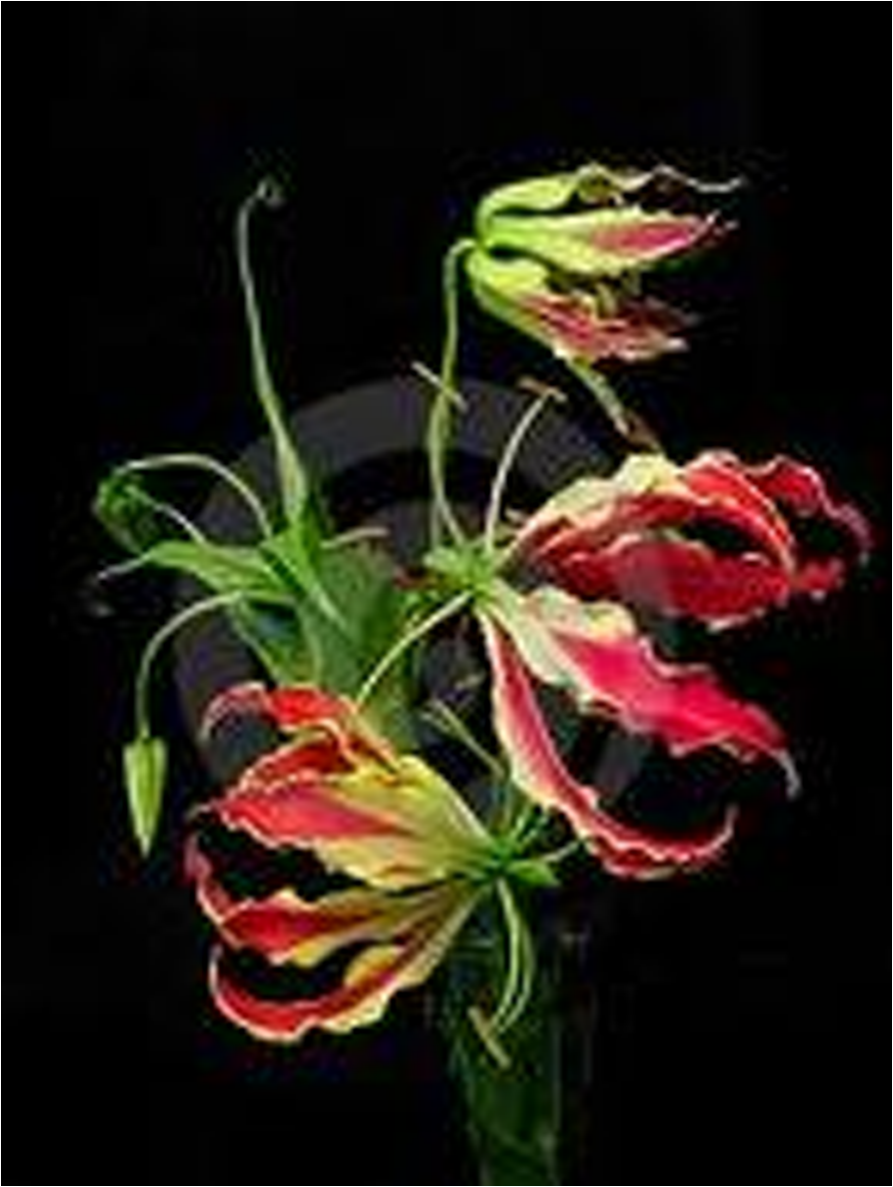
Gloriosa flower

## Conclusion

This patients initial presentation suggested an acute diarrhoeal illness. However by the time he was admitted he had evidence of acute bacterial infection involving the central nervous system, kidneys, lungs and the liver. Although he had rapid defevervescence with antibiotics, there was further deterioration of his illness with clinical evidence of partial intestinal obstruction or paralytic ileus. The possibility of acute leptospirosis, an atypical pneumonia or a meningo enchephalitic illness was considered and empirical antibiotics were commenced. The presence of subconjunctival haemorrhages with gum bleeding and subsequent marked leucopenia with reactive lymphocytosis and thrombocytopenia suggested the possibility of severe comlicated dengue haemorrhagic fever. Although it was not very clear as to why he developed some features of severe leptospirosis or atypical pneumonia during the initial phase of illness, septicaemia complicating acute diarrhoeal phase together with rapid multiorgan involvement of gloriosa toxicity would have been the reasons for fever and neutrophil leucocytosis and for features of central nervous system involvement during the early phase of illness [3, 4]. The subsequent developments that mimicked severe dengue illness are most likely to be due to hepatic and bone marrow toxicity of gloriosa. However, his clinical picture could not be explained with a single possible aetiology and therefore he was managed with extreme care. This patient developed hair loss by day 9 and had massive generalized alopecia by day 14. It was this rapid hair loss within a matter of few days which prompted the likely diagnosis of GSb poisoning as there were no other likely reasons for hair loss such as cytotoxic chemotherapy or treatment with colchicine. The rest of his clinical picture was due to bone marrow toxicity, cerebral, gastrointestinal, hepatic and renal involvement of the toxins of GSb.

This patient did not have any features to suggest cardiac involvement or neuropathy.

In clinical practice, patients who attempt deliberate self harm usually reveal the act at the very begining of the illness. However, this patient divuldged the act of deleberate self harm only after repeated questioning and during the latter stages of his clinical illness. Therefore his illness mimicked an acute severe infection caused by an endemic tropical agent such as leptospirosis or dengue fever until he started to loose his hair. We feel that clinicians practicing in tropical setting where *Gloriosa superba* (Fig. 5) is endemic should be aware of its clinical presentations and should always consider the possibility of ingestion of *Gloriosa superba* when the patient has pancytopenia and develops shedding of hairs which results in total alopecia in a case of unexplained gastroenterocolitis rather investigating [8].

### Ethical statement

We obtained written informed consent from the patient in order to publish his clinical information and the potographic materials without divulging his identity.

## Consent

Written informed consent was obtained from the patient for publication of this Case report and any accompanying images. A copy of the written consent is available for review by the Editor of this journal.

## Abbreviations

ESR: Erythrocyte sedimentation rate
WBC: White cell count
AST: Aspartate transaminase
ALT: Alanine transaminase
CPK: Creatine phosphokinase
CRP: C reactive protein
INR: International normalisation ratio
EEG: Electro enchephalogram
ECG: Electrocardiogram
CT: Computerised tomography
CXR: Chest radiography
USS: Ultrasound scan
IgM: Immuniglobin M
GSb: *Gloriosa superba*
